# Supplementary material for: Analysis of PPARγ Signaling Activity in Psoriasis
Source: Int J Mol Sci. 2021 Aug 10;22(16):8603. doi: 10.3390/ijms22168603 (PMC8395241; doi:10.3390/ijms22168603)
Supplement: Supplementary file 1 [file ijms-22-08603-s001.zip › Supplemental materials_Analysis of PPARg signaling activity in psoriasis/Pathway models/Models images and html files/Interleukin-17 and interleukin-22 signaling in psoriasis/3379.html]

DEFB4A


# Protein DEFB4A

|  |  |
| --- | --- |
| URN | urn:agi-llid:1673 |
| Total Entities | 0 |
| Connectivity | 1330 |
| Name | DEFB4A |
| Description | defensin beta 4A |
| Notes | Defensins form a family of microbicidal and cytotoxic peptides made by neutrophils. Members of the defensin family are highly similar in protein sequence. This gene encodes defensin, beta 4, an antibiotic peptide which is locally regulated by inflammation. [provided by RefSeq, Jul 2008] |
| Class | Transporter |

---

|  |  |
| --- | --- |
| Pathway | Proteins Involved in Acne Vulgaris |
|  | Inflammatory Reaction in Acne Vulgaris |
|  | IL17 Signaling in Psoriasis |
|  | Proteins Involved in Cystic Fibrosis |
|  | Proteins Involved in HIV Type 1 Infection |
|  | Diabetes Induced Periodontitis |
|  | Proteins Involved in Cholesteatoma |
|  | Proteins Involved in Otitis Media |
|  | Proteins Involved in Chronic Obstructive Pulmonary Disease |
|  | Elevated Receptors -> Expression Targets in Mouth |
|  | EGFR -> Expression Targets in Skin |
|  | IFNG/IFNR Expression Targets |
|  | Leptin -> STAT Expression Targets |
|  | Leptin -> ELK/SRF Expression Targets |
|  | EGFR-> Expression Targets in Lymphoid System and Blood |
|  | TNF -> NF-kB Expression Targets |
|  | TNF -> AP-1 Expression Targets |
|  | TNF -> STAT Expression Targets |
|  | TLR3 Expression Targets |
|  | TLR5 Expression Targets |
|  | TLR1 -> 2/6 Expression Targets |
|  | IL1B Expression Targets |
|  | TLR4 -> NF-kB/IRF Expression Targets |
|  | IL1A Expression Targets |
|  | TLR9 Expression Targets |
|  | New Pathway (2) |
|  | Overview. Diseases of the skin (chapter 11) |
|  | 1\_Inflammation in keratinocytes in the pathology of acne |
|  | 2\_Interleukin-17 and interleukin-22 signaling in psoriasis |
|  | 1\_Insufficient activation of immune response in the middle ear epithelium cells in otitis media |
|  | 4\_1\_Polymorphisms associated with inflammatory bowel diseases |
|  | Interleukin-17 and interleukin-22 signaling in psoriasis |

---

|  |  |
| --- | --- |
| Group | Proteins under Expressed in Pyelonephritis |

---

|  |  |
| --- | --- |
| MedScan ID | 1673 |

---

|  |  |
| --- | --- |
| LocusLink ID | 1673 |

---

|  |  |
| --- | --- |
| Alias | defensin, beta 4A |
|  | DEFB4A |
|  | beta defensin 2 |
|  | LOC115252 |
|  | Skin-antimicrobial peptide I |
|  | beta-defensin 4A |
|  | beta-defensin II |
|  | defensin, beta II |
|  | hBD-2 |
|  | DEFB2 |
|  | SAP1 |
|  | Skin-antimicrobial peptide 1 |
|  | beta 2 defensin |
|  | defensin, beta 2 |
|  | DEFB102 |
|  | BD-2 |
|  | DEFB4 |
|  | DEFB-2 |
|  | beta defensin-2 |
|  | defensin, beta 4 |

---

|  |  |
| --- | --- |
| GO ID | 0031731 |
|  | 0042056 |
|  | 0007186 |
|  | 0061844 |
|  | 0019730 |
|  | 0060326 |
|  | 0006935 |
|  | 0050829 |
|  | 0050830 |
|  | 0042742 |
|  | 0006955 |
|  | 0031640 |
|  | 0050918 |
|  | 0005796 |
|  | 0005576 |
|  | 0005615 |
|  | 0045087 |

---

|  |  |
| --- | --- |
| Organism | Homo sapiens {Organism urn:agi-taxid:9606} |
|  | Homo sapiens |

---

|  |  |
| --- | --- |
| OMIM ID | 602215 |

---

|  |  |
| --- | --- |
| Hugo ID | 2767 |
|  | HGNC:2767 |

---

|  |  |
| --- | --- |
| Human chromosome position | 8p23.1 |

---

|  |  |
| --- | --- |
| Swiss-Prot Accession | O15263 |
|  | O15263.1 |

---

|  |  |
| --- | --- |
| GenBank ID | NC\_000008 |
|  | NM\_004942 |
|  | NP\_004933 |
|  | NW\_018654717 |
|  | NG\_023301 |
|  | AC130365 |
|  | AC243982 |
|  | AC270258 |
|  | AF040153 |
|  | AAC33549 |
|  | AF071216 |
|  | AAC69554 |
|  | AJ000152 |
|  | CAB65126 |
|  | BC069285 |
|  | AAH69285 |
|  | BC093983 |
|  | AAH93983 |
|  | BC093985 |
|  | AAH93985 |
|  | BM855542 |
|  | HY264007 |
|  | Z71389 |
|  | CAA95992 |
|  | O15263 |
|  | NC\_018919 |
|  | AMYH02017345 |
|  | AMYH02017346 |
|  | NW\_001841719 |
|  | ABBA01156881 |

---

|  |  |
| --- | --- |
| Ensembl ID | ENSG00000171711 |
|  | ENSP00000303532.2 |
|  | ENST00000302247.2 |
|  | ENSP00000303532 |
|  | ENST00000302247 |

---

|  |  |
| --- | --- |
| Unigene ID | Hs.105924 |

---

|  |  |
| --- | --- |
| Shape | Rhomb |

---
